# Evaluation of Surface Integrity of Multi-Energy Field Coupling-Assisted Micro-Grinding Hastelloy Alloy

**DOI:** 10.3390/mi16050565

**Published:** 2025-05-08

**Authors:** Peng Bian, Zhenjing Duan, Yishuai Jia, Ziheng Wang, Shuaishuai Wang, Ji Tan, Yuyang Zhou, Jinlong Song, Xin Liu

**Affiliations:** State Key Laboratory of High-Performance Precision Manufacturing, Dalian University of Technology, Dalian 116024, China; bian_peng2023@163.com (P.B.); zhenjing456@126.com (Z.D.); jiays2022@mail.dlut.edu.cn (Y.J.); wzh93123456@gmail.com (Z.W.); wangshuais2022@126.com (S.W.); tanji20050125@mail.dlut.edu.cn (J.T.); zyuyang12@mail.dlut.edu.cn (Y.Z.); songjinlong@dlut.edu.cn (J.S.)

**Keywords:** micro-grinding, cold plasma, minimum quantity lubrication, Hastelloy, temperature, surface roughness

## Abstract

Hastelloy is widely used in the manufacturing of high-temperature components in the aerospace industry because of its high strength and corrosion-resistant physical properties, as well as its ability to maintain excellent mechanical properties at high temperatures. However, with developments in science and technology, the amount of available components for use in high-temperature and corrosive environments is increasing, their structures are becoming more complex and varied, and requirements with regard to the surface quality of the components has also become more stringent. The integration of cold plasma (CP) and nano-lubricant minimum quantity lubrication (NMQL), within a multi-physics coupling-assisted micro-grinding process (CPNMQL), presents a promising strategy to overcome this bottleneck. In this paper, micro-grinding of Hastelloy C-276 was performed under dry, CP, NMQL, and CPNMQL conditions, respectively. Contact angle testing, X-ray photoelectron spectroscopy (XPS) analysis, and nano-scratch experiments were used to investigate the mechanism of CPNMQL and to compare the micro-milling performance under different cooling and lubrication conditions employing various characteristics such as grinding temperature, surface roughness, and 3D surface profile. The results showed that at different micro-grinding depths, the micro-grinding temperature and surface roughness were significantly reduced under CP, NMQL, and CPNMQL conditions compared to dry friction. Among them, CPNMQL showed the best performance, with 53.4% and 54.7% reductions in temperature and surface roughness, respectively, compared to the dry condition.

## 1. Introduction

In recent years, with the rapid development of miniaturization and intelligence, the market demand for micro/small parts assembly has been increasing [1,2]. Micro-electronic components, micro-fluidic devices, and micro-mechanical components are widely used in many fields; as a result, microminiature technology continues to grow and develop [3,4,5,6]. Among them, micro-grinding can allow for the avoidance of machining problems such as edges and burrs, improve surface quality, and, at the same time, has the advantages of high efficiency and suitability for difficult-to-machine materials [7]. The tools for micro-grinding use micro-grinding rods with a diameter of no more than 1 mm, and their machining targets are generally fine parts with micrometer dimensions. However, difficult problems, such as the high heat involved in micro-grinding, easy damage of micro-grinding rods, and poor micro-grinding effects on high-temperature alloys, have been limiting the development of micro-grinding technology. Currently, the main direction taken to solve such problems involves researching and designing micro-grinding rods with different materials, structures, and grain sizes, or changing processing parameters [8,9]. C-276 is the most representative grade of Hastelloy, and its internal elements are mainly nickel–molybdenum–iron–tungsten, which belong to a kind of nickel-based high-temperature alloy. C-276 has excellent corrosion resistance and resistance to high-temperatures, and can still have good wear resistance and corrosion resistance in harsh chemical environments and high-temperature environments. These properties are just right for the manufacturing of various types of chemical equipment, such as reactors, heat exchangers, chemical piping, and valves; at the same time, its excellent heat resistance also makes it widely used in the aerospace industry for high-temperature components such as turbine blades, engine combustion chambers, engine exhausts, etc. [10,11,12]. However, because it has the characteristics of high strength and high toughness, it also has problems, such as high grinding temperature, large surface roughness, and too-fast tool wear in grinding processing [13,14,15,16,17,18].

To improve the machinability of the material and the surface quality after micro-grinding, minimum quantity lubrication (MQL) is proposed. MQL atomizes the cutting fluid into micron-sized droplets using a high-pressure air stream and supplies them to the micro-grinding area to reduce friction in the tool–workpiece contact area [19,20,21,22]. However, the cooling performance of MQL is insufficient, and this drawback is particularly noticeable when grinding nickel-based, high-temperature alloys [23,24,25]. Nano-lubricant minimum quantity lubrication (NMQL) is the addition of solid nanoparticles to a base fluid to enhance the cooling lubrication properties of the base fluid, which is then used in the form of MQL [26,27,28]. Nanoparticles have a good heat transfer coefficient, and, when used as an atomizing medium for MQL, the cooling conditions in the micro-grinding zone can be significantly improved [29]. In addition, the nanoparticles are present in the grinding fluid as microscopic solids that resist friction in the grinding zone [30]. High heat transfer coefficients and good friction reduction properties make nano-lubricants important in micro-grinding processes [28]. Talib et al. [31] compared the NMQL system with a conventional cooling system in cutting AISI 1045, and found that NMQL (0.05% h-BN) significantly reduced the cutting forces, cutting temperature, and surface roughness. Gong et al. [32] tested the surface roughness and tool wear for turning Inconel 718 under dry, MQL, and NMQL conditions, which showed that NMQL improved surface quality and reduced tool wear. Yildirim [33] found that MQL has limitations in extreme machining environments, especially in terms of cooling. For this reason, researchers have explored means of mixing nanoparticles into the MQL base fluid to improve the cooling capacity. The nano-lubricant helps to improve the heat transfer and friction characteristics, and it can create an effective oil layer at the tool–workpiece working interface. Meanwhile, Yildirim [34] conducted cutting experiments on high-temperature alloy Inconel 625 under the following three working conditions: dry, MQL, and NMQL, and the results showed that, with the assistance of NMQL, the surface roughness was smaller and the cutting temperature was lower. Sarikaya et al. [35] investigated the effect of nanoparticles of different materials on machining Haynes 25 when used as an auxiliary lubricating medium, and the results showed that hexagonal boron nitride (h-BN) effectively suppressed the notch wear of cutting tools. Korkmaz et al. [36] investigated the effect of various cooling and lubrication methods on machining characteristics where continuous chip formation was observed under MQL and NMQL conditions, and NMQL increased the microhardness of the material surface through the fine particle structure of nanoparticles. Sen et al. [37] milled Hastelloy C-276 under the following three working conditions: dry friction, MQL, and NMQL, and the results of the study showed that, in compared to dry friction, NMQL resulted in a reduction in cutting force, cutting temperature, and surface roughness by 25.49%, 29.84%, and 42.50%, respectively, and a reduction in tool wear by 44.55%.

In addition to MQL and NMQL, cold plasma (CP) has been introduced to cutting processes. Atmospheric pressure CP contains a large number of active particles, with obvious modification effects, which can not change the surface microstructure of the material and significantly improve the material surface wettability [38]. Based on this mechanism, CP generated by gas ionization is proposed to be added to the cutting area as a cooling lubricant medium, referred to as CP-assisted machining. Katahira et al. [39] introduced an atmospheric pressure CP jet in milling silicon carbide diamond and found that the surface roughness was reduced by more than 50% compared to conventional milling at feed rates of 15–40 mm/min. Bastawros et al. [40] added a mixed CP jet of He and H_2_O to the sapphire polishing process and showed a reduction in surface roughness from 1225 nm to 230 nm. Lai et al. [41] proposed a CP-assisted cutting method for machining cast Ce-La alloys, claiming that the arrangement of impurities in the surface layer of the metal can be altered, allowing for improved machinability. Liu et al. [42] suggested that CP can significantly improve the machinability of single-crystal silicon, resulting in lower surface roughness, reduced chip adhesion, and reduced tool wear. Wang et al. [43] conducted experiments on micro-milling metallic glass with the assistance of CP and showed that the roughness was reduced by 37.7–38.2% compared to dry conditions. The above studies have shown that CP is a very significant aid to the metal working process, but the application of this technology in micro-grinding still remains to be further explored.

In summary, the good cooling and lubricating properties of NMQL can reduce the cutting temperature and improve the surface quality of the material; CP can improve the cutting machinability of difficult-to-machine materials and enhance the cooling and lubricating effect of the grinding fluid. However, although the integration of NMQL and CP into a CPNMQL multi-physics coupling-assisted machining approach represents a promising advancement, its applicability and efficacy in micro-grinding processes have not yet been comprehensively validated. This paper presents a nano-lubricant formed by cold plasma-modified h-BN nanoparticles and biodegradable cottonseed oil for MQL, combining with CP to form a multi-field coupling-assisted micro-grinding technique. Micro-grinding experiments were conducted on Hastelloy C-276 under, respectively, four operating conditions: dry, CP, NMQL, and CPNMQL. Wettability tests, nano-scratch experiments, and X-ray photoelectron spectroscopy (XPS) were used to explore the mechanism. The micro-grinding temperature, surface roughness, and 3D surface micro-contour were also used as evaluation indexes to investigate the effect of CPNMQL in assisting the micro-grinding of high-temperature alloys under different working conditions. This provides a new method to improve the surface quality of high-temperature alloys after micro-grinding.

## 2. Experimental Process

### 2.1. Description of Equipment

**Figure 1:**
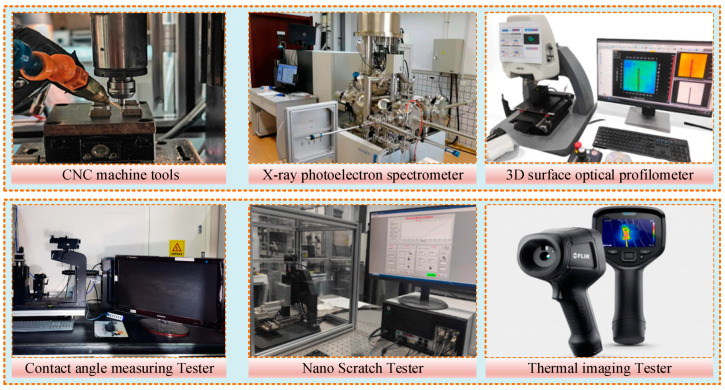
Experimental setup featuring a CNC micro-grinding system, integrated with advanced characterization techniques for comprehensive evaluation of surface integrity.

The experimental CNC machine (EMSF-3060K, Nakanishi, Kanuma-shi, Japan) has a spindle diameter of 30 mm, a maximum speed of 60,000 rpm, a tolerance range of 1 µm, and a maximum spindle output of 350 W. The fuel consumption of the NMQL system (KS-2107, Shanghai Jinzhao Energy-saving Technology Co., Ltd., Shanghai, China) can be continuously adjusted in the range of 0–20 mL/h. The CP system is mainly composed of the following three parts: a power supply system, an electrode system, and a gas source system. The CP power supply (CTP-2000K, Nanjing Suman Electronics Co., Ltd., Nanjing, China) has an output voltage of 30 kV, a rated power of 500 W, and a frequency selection range of 1–100 kHZ. Surface elemental changes after CP treatment were determined by XPS (ESCALAB Xi+, Thermo Scientific, Swindon, UK). A contact-angle-measuring instrument (SL200KS, KINO, Boston, MA, USA) was applied to measure the wettability of the surface of the CP-treated material. Nano-scratching experiments were carried out employing a nano-scratcher (TI 950 TriboIndenter, BRUKER, Shah Alam, Malaysia). The micro-grinding temperature was measured by an infrared thermometer (FLIR E6xt, Pumeng technology, Shanghai, China) during the micro-grinding process. A 3D surface optical profiler (Zygo-9000, ZYGO Inc., Middlefield, CT, USA) was used to observe the microscopic morphology of the material surface and measure surface roughness, and the measurement standard was completely in accordance with the surface shape of ISO 25178 [44]. The specific experimental and measurement equipment is shown in Figure 1.

### 2.2. Workpiece and Tool

The micro-grinding workpiece material was Hastelloy C-276, the composition of which and basic physical properties (room temperature) are shown in Table 1 and Table 2. The workpiece is 10 × 10 mm in size and 5 mm thick and was ground by side micro-grinding. The diameter of the grinding head of the micro-abrasive rod is 0.8 mm, the diameter of the shank is 4mm, the tool substrate used in the micro-grinding experiments was made of CBN (Cubic Boron Nitride) and coated with SDC (Synthetic Diamond Crystal) particles, and the grain size is 320#, which is manufactured by a sintering process.

### 2.3. Preparation of Nano-Lubricant

**Figure 2:**
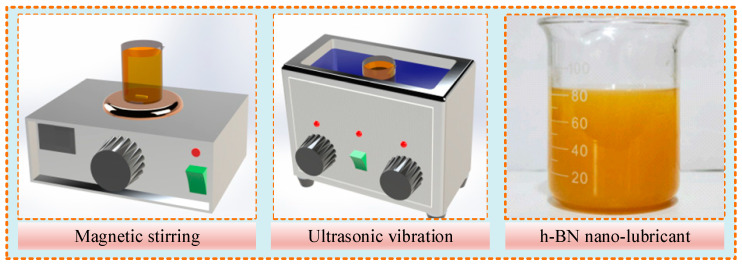
Preparation of h-BN nano-lubricant.

The nano-lubricants used in NMQL were prepared by mixing biodegradable cottonseed oil with CP-modified h-BN nanoparticles with a diameter of 50 nm. The nano-lubricants were produced by stirring cottonseed oil, and 0.3%wt of CP-modified h-BN nanoparticles in a blender for 30 min, then using ultrasonic shaking for 1 h, and finally stirring for another 30 min [45]. The preparation process is shown in Figure 2. CP-modified nanoparticles can increase their dispersion stability in the base solution. The mechanism is to modify the nanoparticles by CP treatment to increase the surface energy of the nanoparticles and improve the wettability of the base solution on the surface of the nanoparticles, which in turn leads to a better dispersion of the nanoparticles in the base solution [46].

### 2.4. Experimental Procedures

#### 2.4.1. Nano-Scratch Experiment

The nano-scratch experiment is essentially a micro-grinding process in which a single diamond grit removes material and simultaneously removes complex interactions between multiple abrasive grains, chips, etc. It can more realistically simulate the mechanism of the action of abrasive particles on the workpiece surface in micro-grinding [47]. To investigate the effect of CP treatment on the removal rate of Hastelloy C-276, loading force nano-scratch experiments were, respectively, conducted on the surfaces of workpieces without CP treatment and after CP treatment. The experimental parameters are shown in Table 3.

#### 2.4.2. Hastelloy C-276 Micro-Grinding Experiments

The main objective of this experiment was to investigate the law of influence on the micro-grinding temperature and surface quality of Hastelloy C-276 under dry, CP, NMQL, and CPNMQL conditions. The specific experimental parameters are shown in Table 4 and Table 5.

## 3. Results and Discussion

### 3.1. Wettability

**Figure 3:**
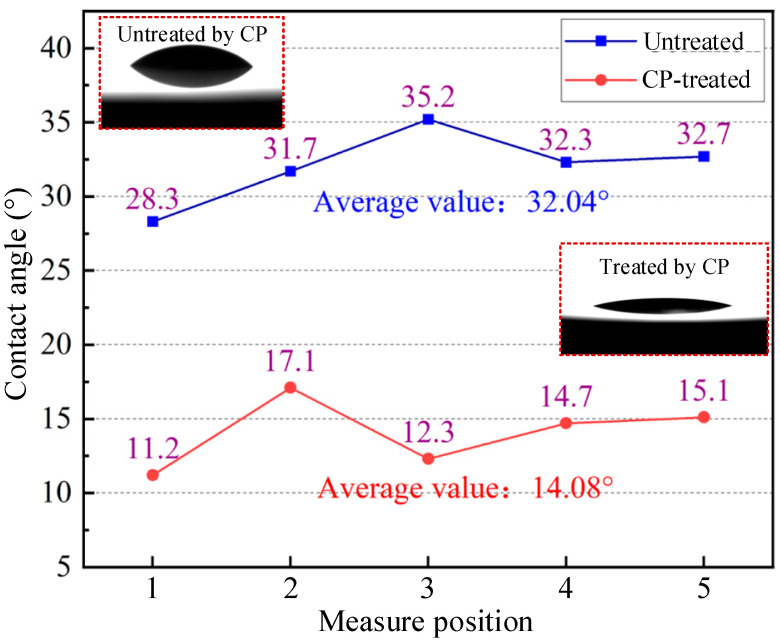
Contact angles of h-BN nano-lubricant on workpiece surface.

Wettability is the ability of liquid to adhere and expand on the surface of solid when the liquid and solid are in contact. It is widely recognized that contact angle is an effective indicator to characterize the goodness of solid–liquid wettability. The smaller the contact angle, the more affinity between the solid and the liquid [48,49]. Similarly, in the machining process, if the cutting fluid wettability is higher, it means that the cutting fluid can better contact the surface of the workpiece effectively, and lubrication and cooling performance are also better [50,51,52]. To investigate the role of CP on the surface wettability of Hastelloy C-276, two sets of comparison experiments were designed, on no-treatment and CP-treated surfaces over a 30 s period. Subsequently, equal drops of h-BN nano-lubricant were applied to five randomized workpiece areas before and after CP treatment, and the magnitude of the contact angles were measured separately. The measurement results are shown in Figure 3. The average value of the contact angle between h-BN nano-lubricant and the untreated workpiece surface is 32.04°, and the mean value of the contact angle after 30 s of CP treatment was 14.08°. It is concluded that CP treatment can significantly improve the wettability of the workpiece surface.

### 3.2. XPS Analysis of Workpiece Surfaces

**Figure 4:**
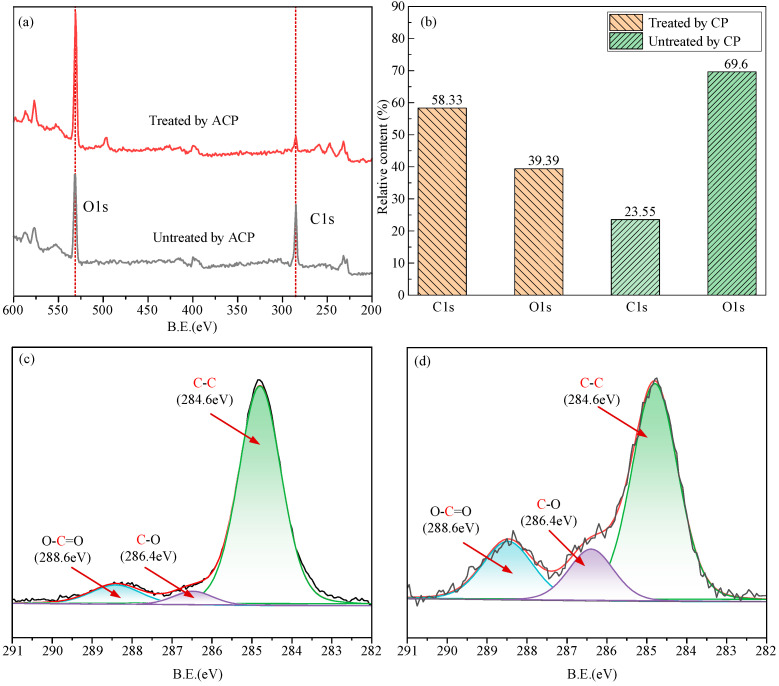
XPS analysis (**a**) XPS spectra; (**b**) relative content of C and O; (**c**) peak-fitted C1s XPS spectra of the CP-untreated surface; (**d**) peak-fitted C1s XPS spectra of the CP-treated surface.

Solid surface energy is one of the key factors affecting the wettability of solid surfaces. When formed on the surface of a substance, due to the different environments that exist between molecules in the surface layer and molecules in the bulk phase, the surface particles have a higher potential energy relative to the inner fabric particles, and this extra energy is called the surface energy [53]. Oxygen ions have unique hydrophilic and reactive oxygen properties, which can significantly enhance intermolecular interactions, thereby increasing surface energy [54]. As shown in Figure 4a, the ratio of O/C elemental content of the workpiece increased significantly after CP treatment. According to Figure 4b, the O content increased from 39.39% before CP, untreated, to 69.6% after CP treatment; C content, on the other hand, decreased from 58.33% to 23.55%. O often exists in the form of oxygen-containing functional groups such as hydroxyl, alcohol, and ester. After high-resolution spectroscopic examination, it was found that the peaks of oxygen-containing groups (C-O, O-C=O) increased after CP treatment, as shown in Figure 4c,d. The increase in the concentration of oxygen-containing functional groups can effectively increase the surface energy of the material, thus increasing the wettability between the workpiece-cutting fluid to achieve better cooling and lubrication.

### 3.3. Material Removal Rate

**Figure 5:**
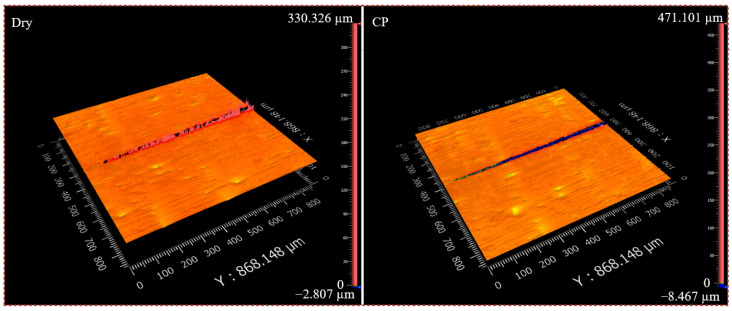
Three-dimensional microstructure of nano-scratches.

**Figure 6:**
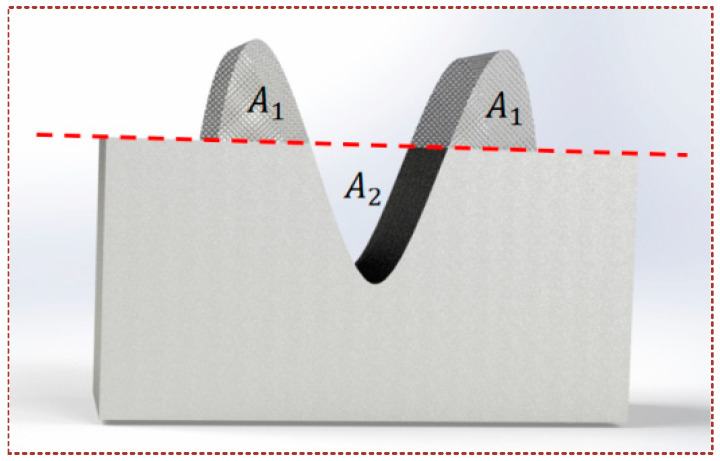
Schematic diagram of scratch section.

**Figure 7:**
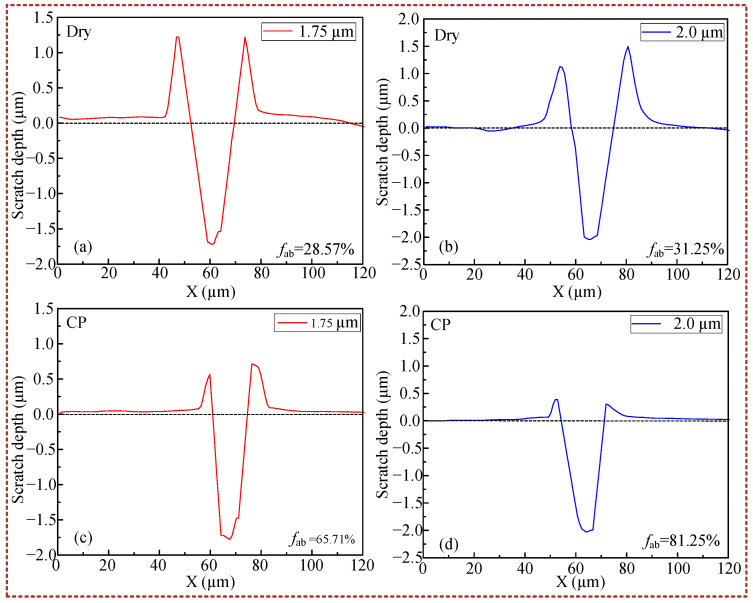
Nano-scratch cross-section: (**a**) scratch depth: 1.75 μm, dry condition; (**b**) scratch depth: 2.0 μm, dry condition; (**c**) scratch depth: 1.75 μm, CP condition; (**d**) scratch depth: 2.0 μm, CP.

The results of the loading force nano-scratch experiment are shown in Figure 5. After nano-scratching of the CP-untreated material, significant plastic ridges appeared on both sides of the scratch. This is due to the plastic deformation of the material and the constant accumulation of abrasive chips that cannot be broken off [55]. And as the loading force continued to increase, the depth of the scratch increased, and the plasticity buildup on both sides also gradually increased. The form of material removal gradually changes from scratching to plowing. However, after nano-scratching of the CP-treated workpiece, the plastic buildup on both sides of the scratch is drastically reduced. This indicates that the CP treatment can significantly reduce the plastic flow on the surface of the workpiece, thus alleviating the plastic buildup phenomenon. To more accurately characterize the effect of CP on the material removal rate, the coefficient *f*_ab_ (Formula (1)) was elicited:

(1) $$f_{ab}=\left(A_{2}-A_{1}\right)*A_{2}^{-1}$$

where *A*_1_ represents the cross-sectional area of the material stack, and *A*_2_ denotes the cross-sectional area of the scratched notch as shown in Figure 6. The *f_ab_* is a value between 0 and 1, when *f_ab_* = 1, pure micro-cutting is the main removal mechanism; when *f_ab_* = 0, it indicates that no material has been stripped from the surface of the material. Therefore, the larger the value of *f_ab_*, the more efficient the removal of the material proves to be. As in Figure 7, at scratch depths of 1.75 μm and 2.0 μm, the calculated values of nano-scratch *f_ab_* without CP treatment were 28.57% and 31.25%. In contrast, when conducting scratch tests under the CP condition, the *f_ab_* calculated values, respectively, were 65.71% and 81.25%. The results show that the material removal rate increases significantly under the CP condition.

### 3.4. Analysis of the CPNMQL Mechanism

**Figure 8:**
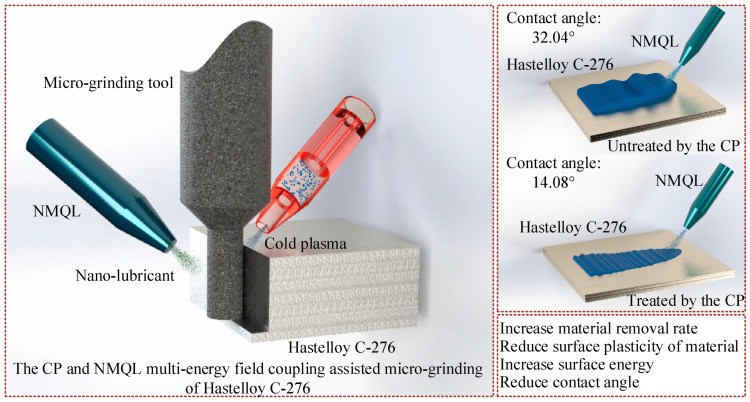
Schematic diagram of CPNMQL-assisted micro-grinding.

As shown in Figure 8, the main effect of CP is to modify the workpiece surface using many active particles under the combined effect of CP and NMQL. The number of oxygen-containing functional groups on the surface of CP-treated Hastelloy C-276 increased dramatically when using XPS, increasing the surface energy of the workpiece, which improved the wetting of the cutting fluid and the workpiece surface. As a result, the nano-lubricant can penetrate better into the micro-grinding area and the contact angle becomes smaller. At the same time, as seen in the nano-scratches experiment, the material removal rate increased.

**Figure 9:**
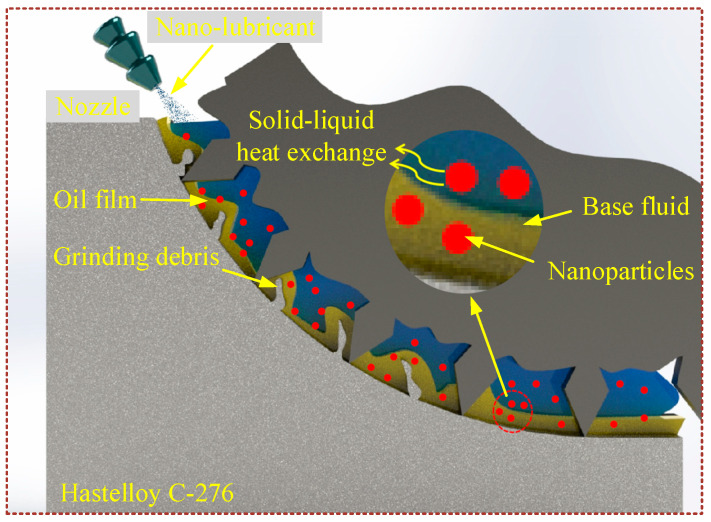
Schematic diagram of nano-lubricant penetration.

**Figure 10:**
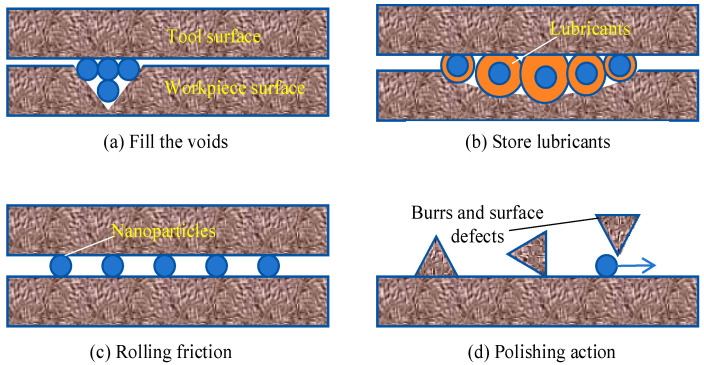
Schematic diagram of nano-lubricant mechanism.

Figure 9 is a schematic diagram of the principle of action of nano-lubricants in the micro-grinding process. First, the nanoparticles have excellent heat transfer properties, and the suspended nanoparticles can form a solid–liquid phase heat transfer with the oil base, eliminating the disadvantage of insufficient cooling performance of the MQL [29]. Secondly, nanoparticles are more likely to penetrate the grinding area during the micro-grinding process and will fill the pits and grooves on the friction interface, preventing direct contact between the interfaces (Figure 10a), and forming a self-lubricating film at the interface, thereby reducing friction and wear [56,57]. At the same time, the presence of nanoparticles is equivalent to increasing the surface area of the grinding zone in another way, which allows more cutting fluid to adhere to the surface of the workpiece, increasing the overall capacity of the cutting fluid [58] (Figure 10b). In addition, the h-BN nanoparticles can act like ball bearings during the friction process, whose effect can effectively disperse the stress between the friction parts, avoiding the concentration of stress to a point and thus causing serious scratches on the surface [59,60] (Figure 10c). The nanoparticles in the nano-lubricant also provide a polishing effect on the surface [61] (Figure 10d).

### 3.5. Temperature

**Figure 11:**
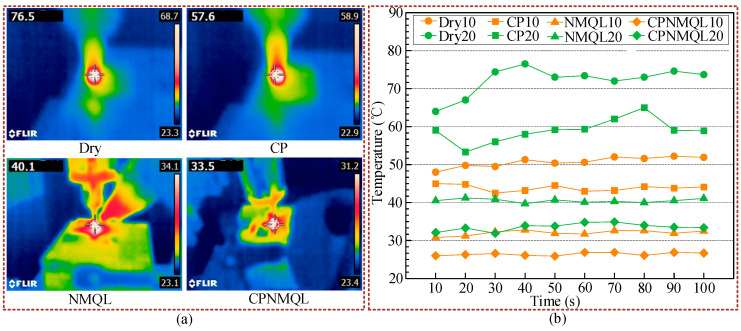
Measurement of grinding temperature: (**a**) infrared temperature measurement process; (**b**) temperature under various conditions.

**Figure 12:**
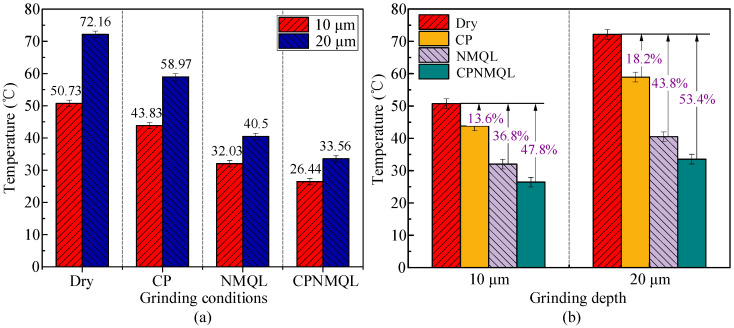
The average value of temperature and comparison of different cooling lubrication conditions: (**a**) temperature comparison under the same cooling and lubrication conditions; (**b**) temperature comparison at the same micro-grinding depth.

In the micro-grinding experiments, an area of 10 mm length is needed on the side-ground, and the feed rate of the micro-grinding head is 100 μm/s, so it takes a total of 100 s to go through working conditions. The experiments were carried out under dry, CP, NMQL, and CPNMQL conditions for two grinding depths of 10 μm and 20 μm, respectively (Figure 11). In the micro-grinding process, an infrared thermometer was used to measure the instantaneous micro-grinding temperature at 10 s intervals, and 10 temperature parameters were obtained for each condition in Figure 11. As shown in Figure 12, the results showed that the average temperature of dry condition and micro-grinding depth of 10 μm was 50.73 °C. At the same micro-grinding depth (10 μm), the average temperatures of CP, NMQL, and CPNMQL, respectively, were 43.83 °C, 32.03 °C, and 26.44 °C. Compared to the dry condition, the temperature was, respectively, reduced by 13.6%, 36.8%, and 47.8%. The average temperature of dry condition and micro-grinding depth of 20 μm was 72.16 °C. At the same micro-grinding depth (20 μm), the average temperatures of CP, NMQL, and CPNMQL, respectively, were 58.97 °C, 40.5 °C, and 33.56 °C. Compared to the dry condition, the temperatures were reduced, respectively, by 18.2%, 43.8%, and 53.4%. It can be found from the data that all three types of assisted micro-grinding (CP, NMQL, and CPNMQL) can reduce the micro-grinding temperature, with CPNMQL having the strongest cooling ability, followed by NMQL and CP having the worst cooling ability. The reason is that the wettability of the CP-treated workpiece surface is improved, and the nano-lubricant can better penetrate the micro-grinding area; the nanoparticles suspended in the lubricant have excellent thermal conductivity and form a good solid–liquid heat transfer channel with the oil base [62]; at the same time, the intense friction and wear in micro-grinding is the essential source of heat, and nano-lubricant has excellent anti-friction and wear reduction effect, so it reduces the generation of heat from the source.

### 3.6. Three-Dimensional Surface Topography

**Figure 13:**
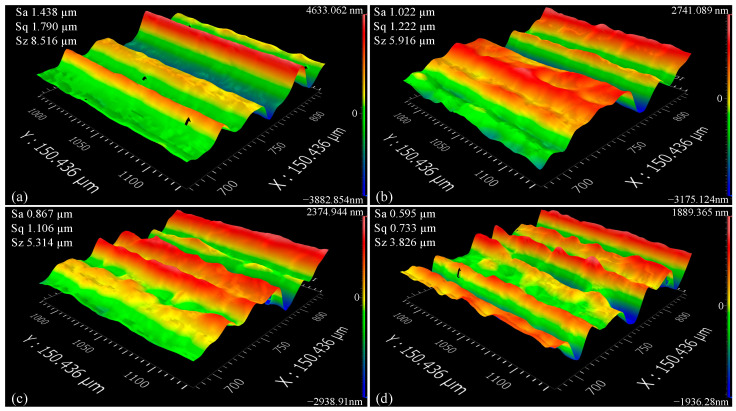
Three-Dimensional surface topography (10 μm): (**a**) dry condition; (**b**) CP condition; (**c**) NMQL condition; (**d**) CPNMQL condition.

**Figure 14:**
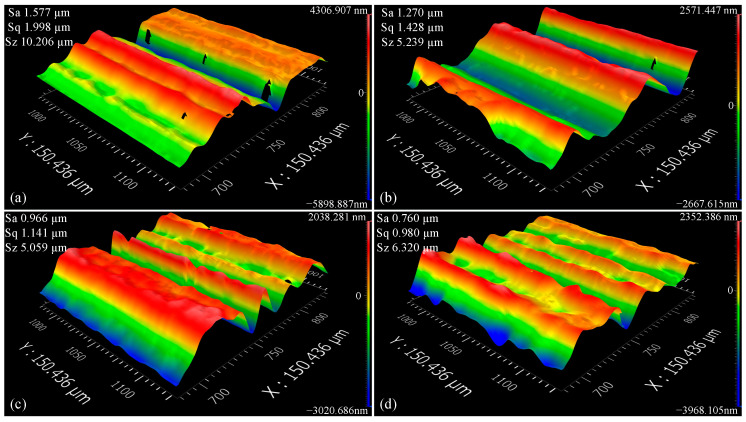
Three-Dimensional surface topography (20 μm): (**a**) dry condition; (**b**) CP condition; (**c**) NMQL condition; (**d**) CPNMQL condition.

As shown in Figure 13 and Figure 14, under CPNMQL conditions, the micro-grinding area is sufficiently cooled and lubricated, and the 3D surface morphology of the workpiece surface has a finer and more compact groove texture arrangement, and the neighboring grooves and protruding peaks are close to each other and have similar shapes. However, under dry and CP conditions, there was no clear pattern in the 3D morphology of the surface, and the gullies were distant and significantly different from each other. When the micro-grinding depths were 10 μm and 20 μm, the groove widths of the surface 3D morphology were generally larger and the shape differences were obvious under dry and CP conditions, and the surface structure was caused by being torn or spalled into micro blocks, which led to the result of larger Sa values. However, under CPNMQL condition, the arrangement of the grooves in the 3D surface topography was finer and more compact with similar shapes, resulting in a decrease in the Sa value.

### 3.7. Surface Roughness

**Figure 15:**
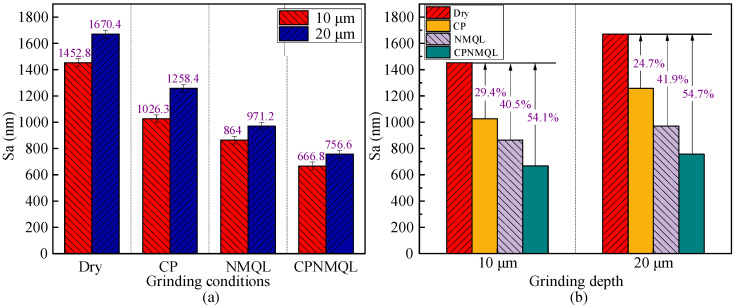
Surface roughness (Sa) values of micro-grinding workpieces under varying auxiliary conditions: (**a**) Sa comparison under the same cooling and lubrication conditions; (**b**) Sa comparison at the same micro-grinding depth.

Sa usually refers to a comprehensive index of surface roughness, which not only considers the surface roughness but may also include some other surface characteristics such as ripple, uniformity, etc. [63,64,65,66]. Sa was measured by Zygo at five equally spaced random locations over a length of 10 mm for each operating condition in Figure 15a. The results show that when the micro-grinding depth is increased from 10 μm to 20 μm, Sa increases in all working conditions. Under the dry condition, the average Sa value increased from 1452.8 nm to 1670.4 nm; the average Sa value increased from 1026.3 nm to 1258.4 nm under CP condition; under the NMQL condition, Sa increased from 864 nm to 971.2 nm; and Sa increased from 666.8 nm to 756.6 nm under the CPNMQL condition. Therefore, NMQL and CPNMQL can effectively mitigate the increase in Sa when the micro-grinding depth increases.

As shown in Figure 15b, when the micro-grinding depth was 10 μm, the Sa of CP, NMQL, and CPNMQL were reduced by 29.4%, 40.5%, and 54.1%, respectively, compared with the dry condition, and when the grinding depth was 20 μm, the Sa of CP, NMQL, and CPNMQL were reduced by 24.7%, 41.9%, and 54.7%, respectively, compared with the dry condition. It can be found that all three assisted methods resulted in a decrease in Sa, with CPNMQL having the most significant effect. The reduction in surface roughness is contributed by a combination of factors. Firstly, CP can increase the material removal rate and wettability of the workpiece surface, and the particles in the nano-lubricant can play multiple roles such as micro-cutting, acting as a bearing, and filling surface defects. With the assistance of such multi-field coupling, the surface roughness of the workpiece can be greatly improved. In addition to this, it is also found that NMQL and CPNMQL make the reduction ratio of temperature and surface roughness both about 40% and 50%, and the reduction shows a clear positive correlation trend. Many studies have proved that grinding temperature is an important factor affecting surface roughness [67].

## 4. Conclusions

In this experiment, micro-grinding of Hastelloy C-276 was carried out under the following four working conditions: dry, CP, NMQL, and CPNMQL. Micro-grinding performance was verified by grinding temperature, surface roughness, and surface 3D morphology for each operating condition. Meanwhile, the action mechanism of CP and NMQL was explored through contact angle measurement, XPS analysis, and nano-scratch experiments. The main findings are as follows:

(1) CP can improve the material removal rate during micro-grinding, increase the surface energy of the material, and thus promote the penetration and adhesion of h-BN nano-lubricants.
(2) CPNMQL can significantly reduce the grinding temperature during the grinding process. Compared with the dry condition, the grinding temperature was reduced by 13.6%, 36.8%, and 47.8% for CP, NMQL, and CPNMQL conditions, respectively, at a grinding depth of 10 μm; and the reduction rates were 18.2%, 43.8%, and 53.4%, respectively, at a grinding depth of 20 μm.
(3) Compared with dry micro-grinding, the average values of Sa under CP, NMQL, and CPNMQL conditions were reduced by 29.4%, 40.5%, and 54.1%, respectively, at a micro-grinding depth of 10 μm; and by 24.7%, 41.9%, and 54.7%, respectively, at a grinding depth of 20 μm. CPNMQL significantly can reduce the surface of the micro-ground workpiece roughness and significantly improve the surface quality.
(4) Calibration of the CP and NMQL nozzle positions is a tedious and slow step during experiments. Manual calibration makes it difficult to achieve the same nozzle position over multiple experiments. This can lead to fluctuations in the measured data. This will reduce the processing efficiency of CPNMQL-assisted machining.

## Figures and Tables

**Table 1:** Elemental composition of Hastelloy C-276.

Element	Ni	Mo	Cr	Fe	W	Co	Mn	V	Other
Component (%)	58.06%	15.38%	15.10	5.69%	3.45%	1.22%	0.68%	0.14%	0.28%

**Table 2:** The physical, mechanical, and thermal properties of Hastelloy C276 alloy.

Properties	Value	Unit
Density	8.89	g/cm^3^
Elastic modulus	205	GPa
Poisson’s ratio	0.31	/
Tensile strength (ultimate)	785	MPa
Yield strength (0.2% offset)	365	MPa
Elongation	59	%
Hardness	87	HRB
Specific heat	427	J/kg°C
Thermal conductivity	10.5	W/m°C
Melting range	1323–1371	°C

**Table 3:** Loading force nano-scratching experiment.

Scratch Speed	Scratch Length	Force Loading Rate	Maximum Force	Time
10 μm/s	1000 μm	10 mN/s	1000 mN	100 s

**Table 4:** Cutting parameters of micro-grinding Hastelloy C-276 alloy.

Spindle Speed (r/min)	Cutting Speed (m/s)	Feed Speed(μm/s)	Micro-Grinding Depth (μm)	Micro-Grinding Width (mm)	Conditions
30,000	1.3	100	10 20	1	Dry CP NMQL CPNMQL

**Table 5:** Parameters of NMQL and CP.

Parameter	Value
Nozzle distance of NMQL (mm)	15
Flow rate of NMQL (mL/h)	8
Air pressure of NMQL (MPa)	0.5
N2 flow of CP (mL/min)	12
Power frequency of CP (kHz)	59.6
Discharge voltage of CP (kV)	1.86

## Data Availability

The original contributions presented in the study are included in the article, further inquiries can be directed to the corresponding author.

## Abbreviations

The following abbreviations are used in this manuscript:

CP	Cold plasma
NMQL	Nano-lubricant minimum quantity lubrication
CPNMQL	Cold plasma and nano-lubricant minimum quantity lubrication
XPS	X-ray photoelectron spectroscopy
MQL	Minimum quantity lubrication
CNC	Computerized Numerical Control
